# On Compressing the Blood-Vessels in Diseases

**Published:** 1812-11

**Authors:** 


*To the Editors of the Medical and Physical Journal.*

GENTLEMEN,

IF the following desultory remarks on several diseases meet your approbation, you are at full liberty to insert them in your very valuable publication.

When lately perusing a small treatise of Mr. George Kellie, surgeon of the navy, relative to the application of tourniquets to the extremities as remedies in various diseases, but particularly in intermittent fevers, I was much astonished with the singular effects thereby produced.

Applied to the extremities during the cold stage of an intermittent, the hot stage soon succeeds, and the paroxysms, upon the whole, are much shortened.

Mr. Kellie undoubtedly deserves much credit for profiting by the hint given him by a Dutch fisherman, relative to the use of ligatures applied to the extremities in agues. Most people would have treated such information with contempt, but it would seem Mr. Kellie's laudable zeal for professional improvement, induces him to collect information from every possible source.

However much I admire Mr. Kellie's ingenuity, I cannot agree with him relative to the *modus operandi* of the remedy alluded to. He says, that, in consequence of the application of tourniquets to the extremities, an unusual plethora is occasioned throughout the rest of the system; and that this preternatural plethora gives rise to an increased vigor of the circulation, which occasions a consequent abbreviation of the cold stage.

Now, I am of opinion, that an unusual plethora of the system does not take place under such circumstances; nay, that the contrary must always be the case to a greater or less degree.

Let it be supposed, that the circulation in one extremity is stopped by a tourniquet. As the compression of the superficial veins must precede that of the principal artery, it is evident that the extremity to which the tourniquet is applied will contain more blood than it did previous to its application. Because, after the superficial veins are compressed, a certain quantity of blood necessarily passes into the extremity by the larger arteries, before they can be compressed by the tourniquet. Besides, it appears to me highly probable, that, during many of Mr. Kellie's experiments, the arterial trunks of the extremities were not altogether compressed. Hence, in such a condition, a very considerable accumulation of blood would take place in those limbs limbs on which the tourniquets were applied. Consequently, the system at large would be deprived of a proportional quantity of blood during the experiment, and therefore, instead of being unusually plethoric while the tourniquets were applied, the system at large would unavoidably be in an opposite condition.

How to account for the increased vigor of the circulation that soon succeeds the application of the tourniquet, I own I cannot altogether satisfy myself. Is it, that, during the cold stage, an unusual resistance is made to the heart, owing to the accumulation of blood in the larger vessels, while the vigor of the heart itself is diminished from the debility then existing, and, being deprived during the application of the tourniquets of a certain quantity of the circulating mass, the heart is then able to propel the remainder with greater velocity? Or, is it that the arteries, which convey the blood to the extremities to which the tourniquets are applied, become unusually distended with blood between the tourniquets and heart, and thus, by the stimulus of over distension, are excited to unusual efforts, and from consent the heart and whole sanguiferous system undergo a similar change? I confess the last opinion seems to me most probable. If the former opinion were correct, the abstraction of blood during the cold stage of an intermittent should shorten its duration. But I am apprehensive that such a practice would produce a contrary effect.

The state of the pulse after amputation of the extremities and operations for aneurisms, seems to invalidate the doctrine now advanced. Indeed, after the operation for aneurism, a preternatural plethora will exist throughout the whole system, (except the limb on which the operation was performed,) greater or less according to the nature of the aneurism.

Suppose the femoral artery to be tied, the blood of the corresponding extremity will be returned to the system by the veins, while, at the same time, the quantity of blood transmitted to the extremity in question by the anastomosing branches of the artery, for several days after the operation, will probably be much less than was transmitted to it previous to the operation; therefore an unusual plethora will exist in the other parts of the system, until the limb on which the operation has been performed is capable of receiving its usual quantity of blood. In this case two powerful causes of exciting the heart and arteries take place; namely, preternatural plethora, and the unusual distension of the trunk of the artery which supplies the diseased limb with blood. Hence, after the operation for aneurism, a greater increased vigor of the circulation should take place than after amputation, every thing else being equal, and, if I mistake not, the fact really is so. I lately saw a person who had undergone the operation for aneurism above Poupart's ligament. Notwithstanding he was much emaciated, the heart and arteries contracted with great violence. Hence, if a person in health undergoes the operation for aneurism, copious blood letting would seem proper.

Reflecting on this subject, I was naturally led to consider the changes produced on the system by different modes of commanding the circulation during amputation of the extremities. If a person is not much emaciated, the present mode used in England of commanding the circulation of the member to be amputated, seems to be best; because, by the operation, an unusual plethora of the system is not produced, but, on the contrary, the system is always somewhat deplenished, and the danger of the subsequent fever consequently diminished. But, if the subject should be much emaciated, would it not be better to compress the artery supplying the limb with blood for five or ten minutes before the application of the tourniquet? The artery ought to be compressed higher than where the tourniquet is to be applied, and the compression should be continued until the tourniquet has been tightly screwed: thus, before the operation, the greater quantity of the blood that existed in the limb to be amputated will return to the system at large; consequently the system will be more plethoric after than before the operation, and in this way additional vigor might be imparted to the system, as if it were by the transfusion of blood.

However, if the subject to be operated on enjoyed tolerable health, and had not previously lost much blood, the compressing the artery in the manner alluded to might occasion a dangerous state of plethora, and consequently violent fever.

It is, I believe, generally allowed by our military surgeons, that the French lose a greater number of men in proportion, who have undergone amputation of the extremities in consequence of recent wounds, than we do. As the manner of operating and dressing the wounds in France and England does not differ materially, it seems to me that the unusual plethora occasioned by the French manner of operating, is the cause of the difference of success. If every thing else were equal, a greater proportion of French should recover than of our men, as the former are less plethoric and more temperate. The French surgeons, for a number of years, have generally amputated the extremities without tourniquets.

tourniquets. The subclavian or femoral arteries are compressed by the thumb of an assistant during the operation; hence a great proportion of the blood which was contained in the limb about to be amputated, is returned to the system at large, and thus may give rise to dangerous plethora and violent fever. Might it not be adviseable to try the effects of compressing the superficial veins of the lower extremities by tourniquets or ligatures, applied near to the groins, in inflammations of the abdomen, chest, or head, or even in apoplexy?

If, while the superficial veins are compressed, the circulation in the femoral arteries continued uninterrupted, it is evident that a very considerable accumulation of blood would soon take place in the lower extremities. It is impossible to ascertain precisely the amount of the additional quantity of blood thus accumulated in the lower extremities; however, it is probable that it would amount, in most instances, to several pounds. Hence, I think it is likely, that depriving the system in general of several pounds of blood for a given time, in the manner now stated, might produce powerful changes on the system at large, probably somewhat similar to those produced by blood-letting; and if, during the operation alluded to, inflammatory diseases previously existing became less violent; then strong presumptive proof would be furnished that additional blood-letting, with the frequent repetition of compressing the superficial veins of the lower extremities would prove beneficial. The afflux of blood to the lower extremities might also be increased by immersing the feet in warm water. As, during this operation, the reflux of blood to the heart by the deep-seated veins would not be prevented, it seems highly probable that the compression of the superficial veins might be continued a very considerable length of time without giving rise to any injurious derangement of the system, either local or general.

As, previous to an apoplectic attack, symptoms of general debility almost always occur, the propriety of large and repeated bleedings seems to me very doubtful. If, after the first bleeding, an amelioration of symptoms took place, a second bleeding might perhaps be advisable; but, if no change for the better should have occurred, and more particularly if the disease should have augmented, additional blood-letting appears to me a very uncertain remedy. Indeed, on reading the history of apoplectic cases, I have often thought that the more copious and frequent the bleedings have been, the more rapid was the fatal termination of the disease. In peripneumonia notha, when the system labors under great general debility, the disease is often rapidly increased, and the patient hurried to his grave by blood-letting.

I own I have not had much experience in apoplexy, yet I have occasionally seen the disease.

Not many years ago, a person of rather a plethoric habit, about thirty years old, and much addicted to drinking spirituous liquors, was found in bed speechless and motionless, his eyes insensible to the impression of light, his breathing stertorous, and pulse slower than natural. He was placed immediately in a chair, and his feet were immersed in warm water. In the course of half an hour he could swallow, and soon afterwards he spoke. Twelve grains of calomel were given him as soon as possible, and he was ordered frequent doses of a mixture containing spiritus minderery. After his feet had been an hour in warm water, he was placed in bed with his head raised, he perspired freely during the night, and next morning he was free from complaint. The calomel operated downwards several times; his drink consisted of weak broth and warm wine and water. The account this person gave of himself was, that he was from home on business, and on his return, finding himself very cold and weak, he went to bed, but was then perfectly sober. Several weeks afterwards he had a similar attack, was immediately bled copiously by the medical man who first saw him, and in two days thereafter he died. Hence it seems probable that bleeding was injurious in this instance.

Some time since I saw an elderly man laboring under a violent attack of apoplexy. He had lost the power of motion, speech, and deglutition. His breathing was stertorous, his eyes were insensible to the impression of light, and his pulse rather slower than natural, but regular. For some days prior to the attack he had head-ach, and at times slight bleeding from the nose, and he was suddenly taken ill in the night. He was naturally extremely healthy, of a florid complexion, and not addicted to drinking to excess. The medical man who attended him ordered a stimulating injection, but would not allow him to be bled; yet, in the space of two days, he began to swallow, and soon afterwards recovered the power of speech and motion in a slight degree. What the result of this case was I know not, as I shortly afterwards left the place where this patient resided, and have not heard of him since.

Although, on dissection of those who die of apoplexy, effusions of blood are generally discovered in the ventricles, substance, or on the surface of the brain, and in recent cases appearances of unusual plethora in the neighbourhood of the effused blood are constantly observed; still it appears to me me absolutely certain, that in proportion as an unusual accumulation occurs in one part of the brain or in its membranes, so the other parts contained within the cranium must be deprived of a proportional quantity of blood; because it is not possible that there can be more blood within the cranium at one time than another: if that were the case, it must be allowed either that the substance of the brain is compressible, or that a vacuum could occasionally exist within the cranium. The absurdity of this opinion is evident; and I believe that it is generally admitted by anatomists, that the substance of the brain itself is incompressible: hence apoplexy must either be produced by pressure applied to the brain, by the over distension of its proper vessels by blood, or by effusion of blood within the cranium; and it is probable that the latter is generally a consequence of the former; therefore, in the incipient stages of apoplexy, it is probable that the blood may often be contained within its proper vessels. On this account, the most powerful remedies should be had recourse to as soon as possible, with a view to prevent the effusion of blood, as the blood-vessels of the brain, when over distended to a certain degree, will at length be excited to unusual actions, and active inflammation and consequent effusion will most likely ensue.

The first case stated in this paper furnishes strong presumptive proof that the symptoms of apoplexy may exist without any effusion of blood.

The predisposing cause of apoplexy seems to be general debility and relaxation; its immediate cause, pressure of the brain, or that derangement in its functions produced by an over distension of its vessels, or an effusion of blood somewhere within the cranium.

In the more advanced periods of life the whole system becomes debilitated, the heart contracts less frequently and less forcibly than in youth, and the blood-vessels throughout the whole system do not propel their contents with their wonted vigor; hence the blood does not flow towards the surface of the body with the same vigor as formerly, and, consequently, the bulk of the external parts frequently gradually diminish; therefore, if a sudden increase of debility should take place, an unusual accumulation of blood will occur in the larger internal blood-vessels, and greater in those parts most plentifully supplied with blood-vessels, and at the same time most relaxed or least capable of opposing resistance.

Hence, reasoning *à priori*, one would suppose the brain, lungs, liver, and other abdominal viscera, most likely to become become the seat of disease in such cases. When the head is thus affected, apoplexy, or a disease of a somewhat similar nature, is the consequence. When an unusual accumulation of blood occurs in the lungs, peripneumonia notha, asthma, or catarrh, ensue, which are frequently succeeded by hydrothorax. And, when the abdominal viscera are affected in a similar manner, chronic inflammations of the liver or spleen are frequently occasioned, and afterwards give rise to ascites.

It may be said, that young people are also subject to sudden general debility, and consequently to preternatural determination of blood towards the internal parts. This certainly is the case, but in youth the vessels are more irritable, and probably also more capable of giving resistance; and consequently do not admit of over distension in such a degree as in the more advanced periods of life. Hence, in young subjects, acute inflammations are most frequent. However, young people who have been much debilitated by long-continued chronic diseases, are liable to complaints similar to those affecting elderly people, &c. &c.

Agreeably to the doctrine now advanced, I should conceive, that, in most cases of incipient apoplexy, much benefit would be derived by producing a determination towards the surface of the body. The capillary vessels throughout the whole system would thus be excited into action; and in proportion as the capillary vessels of the brain were roused to action, the over distension of the larger blood-vessels of this organ would be lessened, and consequently the compression of the brain, arising from this over distension of its larger blood-vessels, would be proportionally diminished.

Although the contractions of the heart have a considerable share in propelling the blood through the larger arteries, yet, I believe, the circulation of the blood in the capillary arteries is carried on in a great measure, if not altogether, by the action of the vessels themselves. I have often compared the action of the capillary arteries to that of leeches when sucking blood; hence, when the nervous energy is much diminished, the circulation of the capillary vessels throughout the whole system is performed very imperfectly. In such a situation there must always be a preternatural accumulation of blood in the larger vessels of the brain, as in proportion as the capillary vessels are deprived of their usual quantity of blood; so a corresponding accumulation must take place in the larger vessels; the quantity of blood within the cranium being at all times the same.

In most instances of apoplexy, I would therefore recommend to put the feet and legs into warm water as soon as possible, possible. If the patient were plethoric, bleeding might then be immediately performed with more safety, as the stimulus of the warm water would counteract in the mean time the debility that might arise from bleeding, and, consequently, might prevent any additional accumulation of blood in the larger vessels of the brain that otherwise might have taken place from the debility induced by bleeding. It also appears to me probable, that ligatures applied to the lower extremities near to the groins, so as to compress the superficial veins, might prove beneficial; as, during this operation, the system at large would be deprived of several pounds of blood, the stimulus arising from the warm water, and also from the over distension of the arteries which supply the inferior extremities with blood, would be rendered more safe. In the mean time, a large dose of calomel and James's powder should be administered, in order to promote perspiration and purging; and light broths and weak warm wine with water should be given as drink, with a view to support the system: thus the action of the capillary vessels would probably be restored and supported; and, if the disease were produced from over distension of the blood-vessels of the brain alone, much relief would probably be soon obtained; but, if effusion of blood had taken place, the benefit would no doubt be much less considerable. James's powder and calomel combined, excite and preserve the action of the extreme vessels better than any medicine I know. Blisters, and other suitable remedies, may be used according to circumstances; and the whole body should be constantly covered with flannel. Tonics, in my opinion, should be administered at an early period of the disease, after proper evacuations have been made.

At all events, I have known several cases treated with considerable success in the manner now described.

*An impartial Medical Man.*

*London, Sept.* 14, 1812.

*Postscript.*—Yesterday, when perusing Mr. Abernethy's excellent work "On the constitutional Origin and Treatment of Local Diseases," I was forcibly struck with the following passage: "I would ask too, practically, does blood-letting cure disorders in which there is a fulness of the vessels of the head? It must be granted, that in many instances it temporarily alleviates them, but in others it fails to relieve, and even aggravates them."

The opinion of Mr. Abernethy deserves the highest attention; as he is a gentleman no less distinguished by those qualities of the mind, which are the greatest ornament of human nature, than by his professional talents.

This quotation seems to be in favor of my opinion on the nature of apoplexy.

Blood-letting relieves inflammations of the trunk or extremities in two ways; namely, by lessening the tension of the sanguiferous system, and likewise by diminishing the impetus of the blood. But, as the quantity of blood within the cranium must at all times be the same, blood-letting cannot immediately lessen the tension of the vessels of a particular part of the brain or its membranes, consequently the benefit derived from bleeding in inflammations of the brain or its appendages, must entirely arise in the first instance from the diminished impetus of the mass of blood at large. Hence, I think, we may in a great measure account for the fatal terminations that generally succeed to acute inflammations of the contents of the cranium. At the same time, it seems highly probable, that the diminished impetus of the blood will give rise to a diminished action of the vessels of the inflamed parts within the cranium, and thus, in the less violent cases, a healthy distribution of the blood within the cranium may be at length gradually restored.

Mr. Abernethy does not specify particularly the diseases that are occasioned by a "fulness of the vessels of the head," neither does he state particularly the nature of the diseases which were temporarily alleviated or aggravated by blood-letting. However, I strongly suspect, that the temporary alleviation of symptoms, which was produced by bleeding, took place in acute inflammations of the membranes of the brain, and that the aggravation of disease from bleeding most frequently occurred in apoplectic cases.

